# Ectopic Posterior Pituitary, Polydactyly, Midfacial Hypoplasia and Multiple Pituitary Hormone Deficiency due to a Novel Heterozygous IVS11-2A>C(c.1957-2A>C) Mutation in the *GLI2* Gene

**DOI:** 10.4274/jcrpe.galenos.2019.2019.0142

**Published:** 2020-09-02

**Authors:** Meliha Demiral, Hüseyin Demirbilek, Edip Unal, Ceren Damla Durmaz, Serdar Ceylaner, Mehmet Nuri Özbek

**Affiliations:** 1 Gazi Yaşargil Training and Research Hospital, Clinics of Paediatric Endocrinology, Diyarbakır, Turkey; 2 Hacettepe University Faculty of Medicine, Department of Paediatric Endocrinology, Ankara, Turkey; 3 Gazi Yaşargil Training and Research Hospital, Clinic of Medical Genetics, Diyarbakır, Turkey; 4 Intergen Genetic Diagnosis Center, Clinic of Medical Genetics, Ankara, Turkey

**Keywords:** Growth hormone deficiency, polydactyly, GLI2 mutations, multiple pituitary hormone deficiency

## Abstract

A novel heterozygous IVS11-2A>C(c.1957-2A>C) mutation in the *GLI2* gene is reported. There was an extremely distinct phenotypical expression in two siblings and their father. The index case was a boy who developed cholestasis and hypoglycaemia in the neonatal period. He had bilateral postaxial polydactyly, mid-facial hypoplasia, high palatal arch, micropenis, and bilateral cryptorchidism. Laboratory examination revealed a diagnosis of multiple pituitary hormone deficiency. There was severe anterior pituitary hypoplasia, absent pituitary stalk and ectopic posterior pituitary on magnetic resonance imaging which suggested pituitary stalk interruption syndrome with no other midline structural abnormality. Molecular genetic analysis revealed a novel heterozygous splicing IVS11-2A>C(c.1957-2A>C) mutation detected in the *GLI2* gene. His father and a six-year-old brother with the identical mutation also had unilateral postaxial polydactyly and mid-facial hypoplasia although there was no pituitary hormone deficiency. This novel heterozygous *GLI2* mutation detected appears to present with an extremely variable clinical phenotype, even in related individuals with an identical mutation, suggesting incomplete penetrance of this *GLI2* mutation.

**What is already known on this topic?**

Patients with *GLI2* mutation usually present with multiple pituitary hormone deficiency (MPHD) accompanied by ectopic posterior pituitary, polydactyly and midfacial hypoplasia. Heterozygous mutations in *GLI2* cause a wide range of clinical phenotypes ranging from asymptomatic cases to more severe clinical phenotypes including Culler-Jones syndrome and holoprosencephaly (HPE) or HPE-like syndrome.

**What this study adds?**

A patient is reported with a novel heterozygous IVS11-2A>C(c.1957-2A>C) mutation in the *GLI2* gene which expands the mutation database. Extremely distinct phenotypical expression and incomplete penetrance of heterozygous *GLI2* mutations may cause MPHD to skip a generation and thus delay or missed diagnosis of these life-threatening hormonal disorders. The response to growth hormone (GH) replacement may be excellent. It is suggested that a trial of GH therapy in cases of *GLI2* mutation with GH deficiency may be beneficial.

## Introduction

The sonic hedgehog (SHH) signalling pathway regulates differentiation, proliferation, tissue polarity, stem cell population, and carcinogenesis of the notochord and floor plate in the developing spinal cord (1,2). The SHH signalling pathway is mediated by three related zinc-finger transcription factors (GLI1, GLI2, and GLI3) which are members of the GLI-Kruppel family. GLI2 is an activating zinc-finger transcription factor which plays a crucial role in the development of the diencephalon and distal extremities during embryogenesis. It is encoded by the *GLI2 *gene, a large polymorphic gene, that is mapped to 2q14.2. Therefore, it is very likely that analysis will show variants of uncertain significance (VUS). Homozygous deletion of both *GLI1* and *GLI2* results in complete absence of the pituitary gland (3). Heterozygous mutations of the *GLI2* gene cause a variety of clinical phenotypes, ranging from asymptomatic cases to more severe clinical phenotypes including Culler-Jones syndrome and holoprosencephaly (HPE) or HPE-like syndrome. Culler-Jones syndrome is a clinical spectrum of multiple pituitary hormone deficiency (MPHD), ectopic posterior pituitary, and postaxial polydactyly with or without midline defects and developmental delay (3). HPE presents with a more severe clinical spectrum with additional midline structural abnormality and forebrain cleavage defects. To date, about 25 different pathogenic *GLI2* mutations have been identified (4). Heterozygous *GLI2* mutations can be inherited in an autosomal dominant fashion or *de novo* (51% maternal, 40% paternal, and 9% *de novo*) (5). Herein, we report a novel heterozygous IVS11-2A>C(c.1957-2A>C) mutation in the *GLI2* gene in two siblings and their father from a non-consanguineous marriage, suggesting an extremely distinct phenotypical expression and incomplete penetrance.

## Case Report

### Index Case

The proband was a male patient who was born after 40 weeks uneventful gestation via spontaneous vaginal delivery, with a birth weight of 3700 gr. The parents were not consanguineous. Family history revealed that one of his brothers, his father and paternal grandfather had polydactyly and atypical facial appearance with no known hormonal disorders. He had postaxial polydactyly, mid-facial hypoplasia, high palatal arch, micropenis and bilateral cryptorchidism. At the age of two months, he developed cholestasis and hypoglycaemic episodes. Growth hormone (GH), cortisol, and insulin concentrations were measured from critical blood samples which revealed a diagnosis of congenital MPHD (Table 1). Hypoglycaemia and cholestasis resolved with replacement of hydrocortisone and sodium L-thyroxine (L-T4). He had severe anterior pituitary hypoplasia, absent pituitary stalk and ectopic posterior pituitary with no other midline structural abnormality on pituitary magnetic resonance imaging (MRI). A surgical orchidopexy was performed. Diagnosis of GH deficiency was confirmed at the age of one year, and GH replacement therapy was commenced at another paediatric endocrine centre.

**Figure 1:**
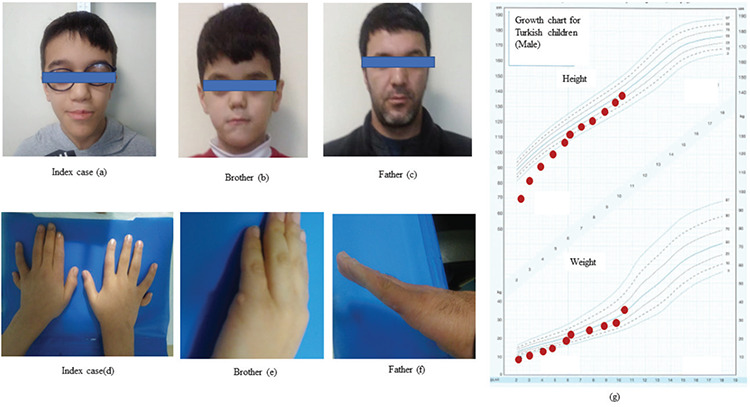
Facial dysmorphism and polydactyly in the index case, brother and father (a-f). Good response to recombinant human growth hormone therapy in the index case (g)

The patient was admitted to our hospital for the first time when he was 2.1 years old. He had been on GH replacement therapy for one year. His weight was 9 kg [-3.3 standard deviation score (SDS)] and height was 69 cm (-5.4 SDS). During follow up at our clinic response to the GH therapy was excellent (see Figure 1). At his most recent follow-up visit when he was 10-years-old, his height was 133.5 cm (-0.46 SDS), weight was 28.7 kg (-0.51 SDS), body mass index was 16.1 kg/m^2^ (-0.4 SDS). He had no signs of puberty. He had bilateral postaxial polydactyly, mid-facial hypoplasia, high palatal arch and moderate developmental delay. He was on L-T4 (2.6 µg/kg/day), GH (with a dose of 0.033 mg/kg/day), hydrocortisone and antiepileptic therapy for focal epileptic seizures.

The patient’s brother was six-years old with a weight of 20.7 kg (-0.01 SDS), and height was 116.2 cm (0.01 SDS). He had normal sized, pre-pubertal testes with no history of undescended testis. He had left postaxial polydactyly and mid-facial hypoplasia with no pituitary hormone deficiency. The patient’s father was 38-years-old and his adult height was 166 cm. He also had left postaxial polydactyly and mid-facial hypoplasia with no pituitary hormone deficiency (Table 1). Cranial MRI was not performed in the father and sibling as they had no evidence of pituitary dysfunction.

### Molecular Genetic Analysis

Genomic DNA was extracted according to the manufacturer’s standard procedure using the QIAamp DNA Blood Midi Kit (Qiagen, Hilden, Germany). All coding exons of the *GLI2 *gene and their flanking splice site junctions were amplified using in-house designed PCR primers (available upon request). These were subsequently sequenced by the MiSeq next-generation sequencing (NGS) platform (Illumina Inc., San Diego, CA, USA). The libraries were prepared with the NexteraXT kit (Illumina Inc., San Diego, CA, USA), according to the manufacturer’s instructions. Next-generation sequencing was carried on MiSeq (Illumina Inc., San Diego, CA, USA). Sequences were aligned to the hg19 genome within MiSeq Reporter software (Illumina Inc., San Diego, CA, USA). The data were visualized with IGV 2.3 (Broad Institute; http://exac.broadinstitute.org/) software. Sanger sequencing analysis was performed for confirmation of the variant detected at NGS analysis.

*In silico* prediction tools (MutationTaster and Human splicing finder) were used for evaluation of the novel unpublished variant. The variant was classified based on the 2015 American College of Medical Genetics and Genomics and Association for Molecular Pathology guidelines (6).

The study was conducted in accordance with the principles of the Declaration of Helsinki and was approved by the Local Ethical Committee. Written informed consent was obtained from the participants and their legal guardians.

**Figure 2:**
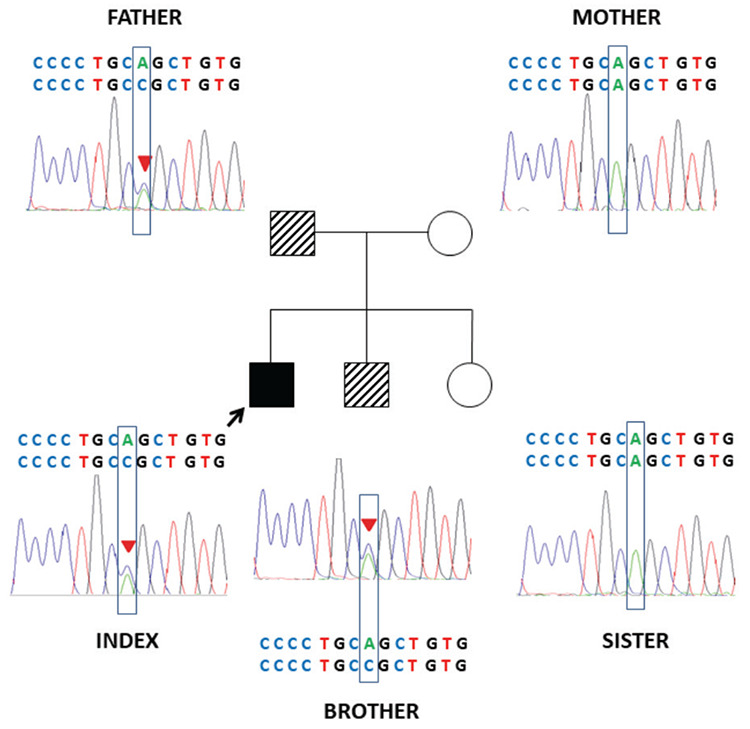
Family pedigree and electropherogram of heterozygous IVS11-2A>C(c.1957-2A>C) mutation in the *GLI2* gene. Full-black filled box indicates index case with Culler-Jones syndrome phenotype, shaded boxes indicate father and brother who are also heterozygous for the identical mutation with incomplete phenotype, empty boxes indicate mother and sister with wild type

A novel heterozygous IVS11-2A>C(c.1957-2A>C) mutation in intron 11 of the *GLI2* gene was identifid in the proband (Figure 2). His father and six-year-old brother, who both had postaxial polydactyly and facial dysmorphism with no hormonal deficiency, were also heterozygous for the identical mutation. The unaffected mother and sister had normal alleles. This variant was listed neither in the 1000 genomes nor in the ExAC database (http://browser.1000genomes.org/index.html, http://exac.broadinstitute.org/, respectively). This mutation in *GLI2* disrupted the intron 11 acceptor splice-site and this was predicted to result in aberrant splicing, and thus synthesis of a truncated protein.

## Discussion

Herein, a patient is presented with congenital MPHD, midfacial hypoplasia, bilateral postaxial polydactyly, anterior pituitary hypoplasia and ectopic posterior pituitary due to a novel heterozygous splicing mutation IVS11-2A>C(c.1957-2A>C) in the *GLI2* gene. Clinical features were similar to Culler-Jones syndrome. Although his father and brother with the identical heterozygous mutation had similar physical dysmorphisms, including postaxial polydactyly and mild facial hypoplasia, they had no hormonal deficiency (Table 2).

The heterozygous IVS11-2A>C(c.1957-2A>C) mutation is predicted to cause a splicing defect that would result in aberrantly spliced transcripts, and thus the synthesis of a truncated protein. *GLI2* mutations leading to a truncated protein usually cause panhypopituitarism, polydactyly and midfacial hypoplasia, which were present in our index case. Interestingly, pituitary dysfunction was not detected in the proband’s father and brother, both of whom had the identical mutation, suggesting incomplete penetrance and variable expressivity (3,5,7,8). Distinct clinical phenotypes in subjects with identical heterozygous *GLI2* mutations have previously been reported and suggested as evidence for incomplete penetrance and variable expressivity (3,9). The variable expression of the *GLI2* gene mutations has been attributed to the combination of genetic, environmental and epigenetic factors or contribution of the other genes involved in the SHH pathway, which include *SHH*, *ZIC2*, *SIX3*, *PTCH1*, *GLI3* and *TGIF* genes (5,9,10,11).

The largest cohort with *GLI2* variants was reported by Bear et al (5) where a *GLI2* variant was detected in 112 of 400 patients with HPE spectrum, endocrine disorders or craniofacial anomaly. Of these 112, 43 were found to have a truncating mutation (frameshift, nonsense, or large deletion) and 69 were reported to have a VUS (5). The clinical characteristics of cases with *GLI2* mutations reported so far are shown in Table 3 (Supplementary file).

The clinical spectrum of mutations in *GLI2* may vary from asymptomatic individuals to polydactyly, functional and structural abnormality in the pituitary gland, facial dysmorphism, Culler-Jones syndrome, HPE-like syndrome, and frank HPE (4,8). In addition, renal problems such as renal hypoplasia/dysplasia, urethral stricture and cardiac problems such as ASD/VSD have been reported in patients with *GLI2* mutations (4,8). HPE is the most common anterior brain anomaly and HPE is characterized by incomplete separation of cerebral hemispheres and underdeveloped midbrain structures. However, the mutations in *GLI2* are rarely associated with an HPE phenotype (7,12). Indeed, in the study of Bear et al (5) only three of the 112 (2.7%) patients with *GLI2* mutations, had HPE (13). Also, neuroanatomical anomalies, such as agenesis of the corpus callosum, abnormal cerebral periventricular venous system and abnormal gyri have been reported in patients with *GLI2* mutations (8,14,15,16,17). In contrast to the literature, our patient had severe anterior pituitary hypoplasia, MPHD, and ectopic posterior pituitary with no features of HPE or HPE like syndrome. Pituitary stalk interruption syndrome (PSIS) is a congenital anomaly of the pituitary gland characterized by small or absent anterior pituitary lobe, interrupted or absent pituitary stalk, and ectopic posterior pituitary lobe (18). PSIS may be associated with isolated or syndromic features (18). Mutations in genes encoding transcription factors in signalling pathways, especially *GLI2 *variants, have been reported in PSIS, which is consistent with our case (18,19).

Pituitary dysfunction due to *GLI2* mutations may vary from idiopathic GH deficiency to MPHD**,** with or without ADH deficiency (3,5). Our index case had biochemical and hormonal features of complete anterior pituitary hormone deficiency including GH, thyroid-stimulating hormone, adrenocorticotropic hormone (ACTH), prolactin, follicle-stimulating hormone (FSH) and Luteinizing hormone (LH) (Table 1). The most common pituitary hormone deficiency is GHD (20). Although the response to rhGH replacement has been reported to be poor in some cases with *GLI2* mutations, an excellent response to rhGH replacement was observed in our case and has been reported previously. This suggests that clinicians should consider a trial of rhGH therapy in cases with *GLI2* mutation who have GHD (Figure 1) (3,8,21). In addition, hypoglycaemia, cholestasis, recurrent seizures and intellectual disability have been reported in patients with *GLI2* mutations as a consequence of ACTH and GH deficiency (22). Hypoglycaemic episodes and cholestasis in our case resolved after replacement of hydrocortisone and with rhGH therapy. We also attributed the seizures and moderate developmental delay evident in our case to neonatal hypoglycaemic episodes due to ACTH and GH deficiency. While the presence of micropenis in our case may be attributed to GH deficiency, he also had cryptorchidism and inappropriately low FSH, LH and testosterone levels during mini-puberty, suggesting concomitant gonadotropin deficiency. Despite having an ectopic posterior pituitary on pituitary-imaging he had no diabetes insipidus at presentation and this has not developed to date during follow-up.

## Conclusion

In conclusion, extra-pituitary findings may provide clues for the diagnosis of particular gene mutations including *GLI2*, *HESX1*, *LHX4*, *SOX3*, and *OTX2 *which are involved in the development and differentiation of the pituitary gland resulting in a variety of pituitary hormone deficiencies. In cases presenting with MPHD accompanied by ectopic posterior pituitary, polydactyly and midfacial hypoplasia, a diagnosis of *GLI2* mutation should be considered. Furthermore, extremely distinct phenotypical expression and incomplete penetrance of heterozygous *GLI2* mutations may be associated with MPHD skipping a generation and thus delay or missed diagnosis of these life-threatening hormonal disorders. In light of this genetic analysis of either asymptomatic or symptomatic relatives for *GLI2* gene mutations and evaluation of carriers for panhypopituitarism is warranted.

## Figures and Tables

**Table 1:**
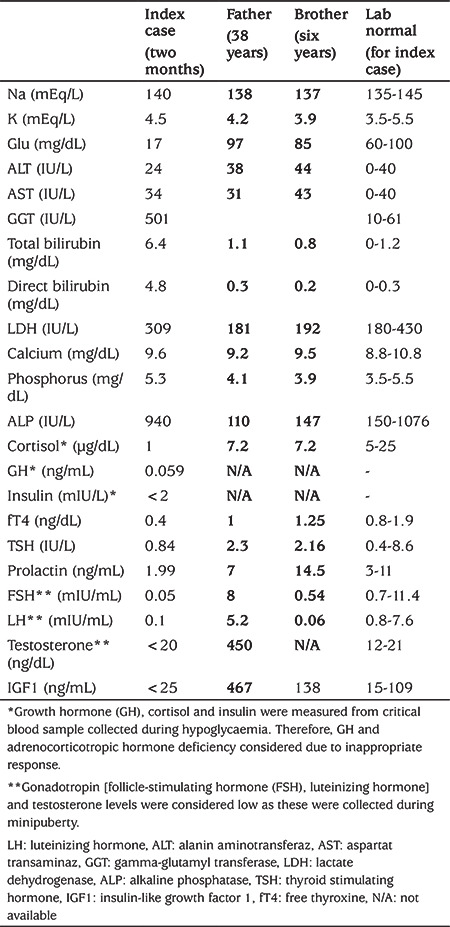
Biochemical and hormonal characteristics of the index case and affected relatives

**Table 2:**
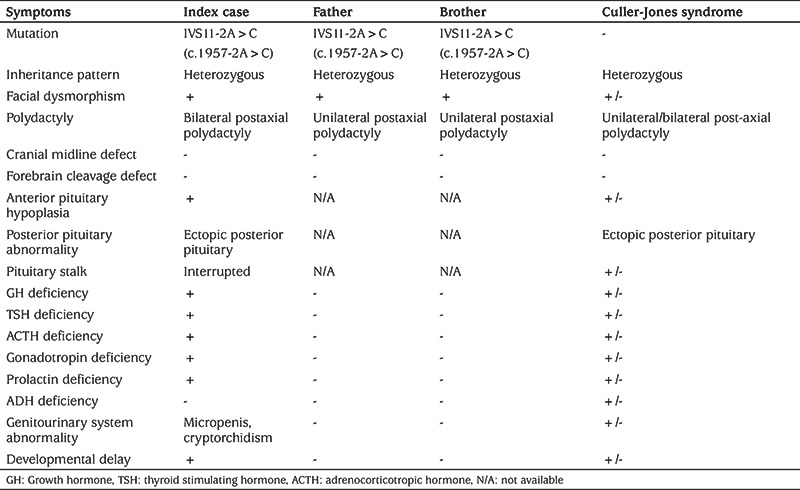
Clinical characteristics of index case were different from father and brother with identical *GLI2* mutation and similar to Culler-Jones syndrome

**Table 3 (Supplementary file):**
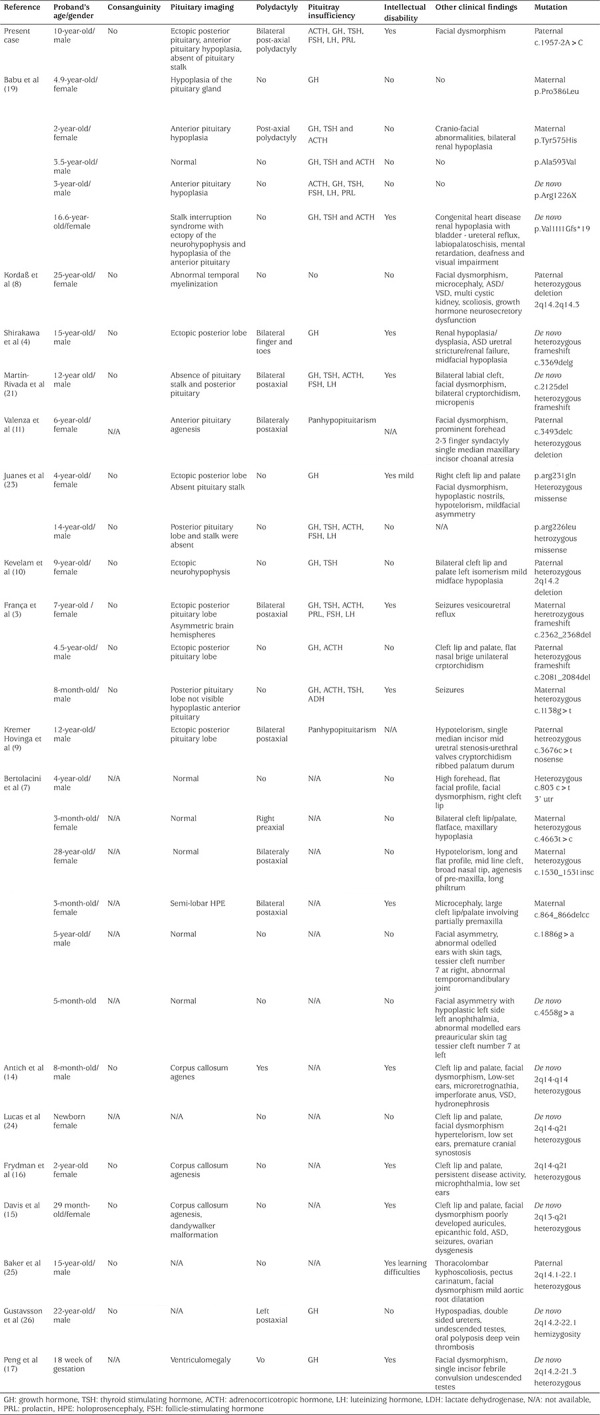
Clinical and genetic characteristics of cases with mutations in *GLI2* gene
